# The Impact of Time Delays on the Robustness of Biological Oscillators and the Effect of Bifurcations on the Inverse Problem

**DOI:** 10.1155/2009/327503

**Published:** 2008-11-02

**Authors:** Nicole Radde

**Affiliations:** 1 Institute for Medical Informatics, Statistics and Epidemiology, University of Leipzig, HÃ¤rtelstraÃŸe 16-18, 04107 Leipzig, Germany

## Abstract

Differential equation models for biological oscillators are often not robust with respect to parameter variations. They are based on chemical reaction kinetics, and solutions typically converge to a fixed point. This behavior is in contrast to real biological oscillators, which work reliably under varying conditions. Moreover, it complicates network inference from time series data. This paper investigates differential equation models for biological oscillators from two perspectives. First, we investigate the effect of time delays on the robustness of these oscillator models. In particular, we provide sufficient conditions for a time delay to cause oscillations by destabilizing a fixed point in two-dimensional systems. Moreover, we show that the inclusion of a time delay also stabilizes oscillating behavior in this way in larger networks. The second part focuses on the inverse problem of estimating model parameters from time series data. Bifurcations are related to nonsmoothness and multiple local minima of the objective function.

## 1. Introduction

The investigation of regulation mechanisms underlying various properties of cellular networks has gained much attention in recent years. Especially interesting in this setting is the relation between the topology of a regulatory network, often referred to as *wiring diagram* or *interaction graph*, and the ability of the system to exhibit certain kinds of dynamic behaviors. It is well known that feedback control mechanisms are essential for phenomena such as hysteresis, bistability, multistationarity, and periodic behavior (see, e.g., [1â€“, 4] or [5] for a more recent review).

While these feedback mechanisms are necessary to capture such phenomena, their existence is usually by no means sufficient. A strong nonlinearity in the Goodwin oscillator model, for example, is a very restrictive requirement for oscillations [6, 7]. Cooperative interaction is also needed to capture switch-like behavior between two or more stable steady states [6, 8â€“, 10]. Thus, the qualitative behavior of the system depends considerably on the exact parameter values [11]. Periodic behavior, for example, can often only be observed for a small fraction in the parameter space, which is bounded by bifurcation manifolds [12].

This is in contrast to real biological systems, which exhibit their function reliably under varying external conditions and internal noise [13â€“, 15], raising the question how this robustness is achieved. The design principles of biological networks are assumed to be a result of a long evolutionary process [16, 17], during which the principles are optimized for a reliable functioning. Signaling networks, for example, have to be sensitive to signals and robust against random perturbations and internal fluctuations at the same time [18â€“, 20]. Many cellular oscillators such as the circadian clock have to maintain a constant period and amplitude under a wide range of different external conditions [21]. The regulatory network underlying the cell cycle has to be robust against perturbations, since dysfunctions may lead to programmed cell death or to phenotypes that are not able to survive for a long time, if at all [22, 23].

All these biological examples investigate a property of organisms which can be described by *functional robustness* [15]. However, the exact definition of robustness varies in all these publications, which indicates that a formalization of the concept of robustness has not yet been established [15, 24]. Further, this goes along with the question about which mechanisms are potentially related to such a robustness.

In this paper, we focus on the robustness of biological oscillator models with respect to varying model parameters. Time-scale differences, time delays, and, related to that, feedback loops comprising a large number of interactions, have already been shown to maintain periodic behavior in chemical reaction systems (see [1, 25] and references therein). Scheper et al. [25], for example, demonstrated the importance of nonlinear regulation and time delays on a model of the circadian oscillator. Chen and Aihara [26] investigated the effect of large time-scale differences and time delays on a two-component oscillator model. Generalizations of their results can be found in [27]. A stabilization of oscillations via time delays among others has also been reported in [21, 28, 29]. While many of the earlier studies refer to two-component systems, interesting recent studies indicate the impact of multiple interlocked feedback loops for the robustness of periodic behavior [30â€“, 35].

This work focuses on oscillations induced by including a time delay into the differential equation model. This inclusion can destabilize a stable fixed point by a Hopf bifurcation. An ordinary differential equation (ODE) model describes the cell as a homogeneous chemical reaction system, assuming that the time between cause and effect of a regulatory process can be neglected. This is of course a simplification, since time delays play a role in many regulation processes. Examples are the transport of mRNA from the nucleus to the cytoplasm, diffusion processes, especially in eukaryotic cells, or the time between binding of a transcription factor to the DNA and the corresponding change in concentration of the regulated gene product. The inclusion of such time delays into the ODE models can change the dynamic behavior of the system qualitatively. Furthermore, the period of oscillations has been shown to be crucially regulated by such a delay [21].

The model class considered here is characterized by monotonicity and boundedness constraints, which will be explained in detail in Section 2.1. This class is similar to systems investigated by Kaufman et al. [10] and Pigolotti et al. [36]. Since the proofs rely on very weak assumptions about the differential equation system, they apply to many two-component oscillator models which have already extensively been studied (see, e.g., [6, 25, 26]). In this sense, the paper generalizes some of the previous publications.

Section 2.2 shows results for two-dimensional systems. In particular, sufficient conditions for the destabilization of a steady state via a time delay are introduced, which imply the existence of a stable limit cycle.

Higher dimensional feedback systems are studied in Section 2.3. For a single negative feedback, system I shows that the inclusion of a time delay can destabilize a stable fixed point through a Hopf bifurcation, implying oscillating behavior. In turn, an unstable fixed point cannot become stable through a time delay.

Section 3 elucidates the problem of robustness of oscillations from a different point of view, the inference of oscillating models from time series data. We will demonstrate on a two-gene network that bifurcations complicate parameter estimation considerably. They are related to nonsmooth error functions with multiple local optima. The special focus in this study is on the bifurcations relevant for chemical oscillator models. As already pointed out by several authors (see, e.g., [37â€“, 40]), results emphasize that advanced parameter estimation approaches for differential equations are required in this context. Finally, conclusions and ideas for future work are provided in Section 4.

## 2. Stabilizing Oscillations with Time Delays

### 2.1. Modeling Biological Oscillators

We consider the following ODE model:(1)

with a continuously differentiable function . The function  is characterized by the monotonicity of each component  with respect to . This condition assigns each regulator  of  either a purely activating or a purely inhibiting function. In the first case,  independent of the state  of the system, the second case corresponds to  for all states . Such systems can be illustrated by directed graphs  with sign-labeled edges, often denoted *interaction graphs*, or, equivalently, by the signed Jacobian matrix  with elements  according to the edge signs in . Properties of this model class and the role of feedback loops are discussed in [1, 2, 4].

Moreover, we assume solutions of the system to be bounded. This is a biologically plausible assumption, but excludes simple linear models. This boundedness constraint is, for example, fulfilled for all network models which describe degradation of network components as a first-order decay process and assume bounds for the production rate [27]. Solutions of these systems have the tendency to converge to steady states [41, 42]. Thus, more complex behavior such as oscillations is typically caused by destabilizing this steady state via Hopf bifurcations [43]. Such a bifurcation requires the existence of a negative feedback loop in  [2]. Hence we focus the analysis onto the investigation of the stability of fixed points , which can be done via investigating the eigenvectors of the Jacobian matrix . Elements of  will be denoted by  throughout the manuscript, dropping their dependence on the coordinates of . According to Lyapunov's indirect method [44], a hyperbolic steady-state  is stable if all eigenvalues  of  have negative real parts [44, 45]. This statement also holds for differential equations including time delays [46]. Further, we will refer to conditions that imply periodic behavior when a fixed point is destabilized.

### 2.2. Stabilizing Oscillations with Time Delays in Two-Dimensional Systems

We consider the following delay differential equation (DDE) system:(2)

with monotonicity and boundedness constraints as defined above. For short, we will use the common notation  for  and  for  subsequently. System 2 is infinite dimensional, since the initial conditions are real-valued functions , which can complicate the analysis considerably. The stability of fixed points of (2) can, however, analogous to ODE systems, be determined by investigating the signs of the real parts of the roots of the characteristic equation. This characteristic equation for a fixed point  of system (2) is derived via linearizing about  (see also [46â€“, 48]) as follows:(3)

with (4)

Calculating the determinant,  is given by(5)

Equation (5) is a polynomial of degree two for , which has two complex conjugate solutions . For , it is a transcendental equation with a countable infinite number of roots. However, the number of roots in the right-half plane is known to be finite [49]. Here, we will only investigate the course of the two solutions  in dependence of .

**Figure 1:**
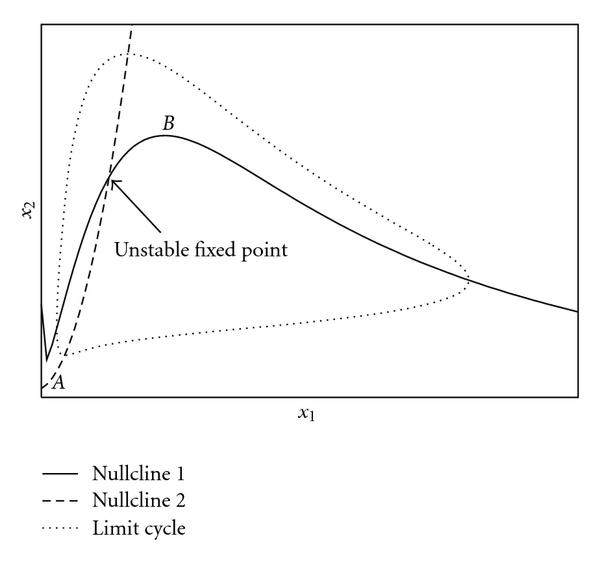
**Nullclines of an activator inhibitor oscillator model.** If the system has a single fixed point  which is unstable, there exists a stable limit cycle around  according to the PoincarÃ©-Bendixson theorem. A necessary condition for  to be unstable is the location between the minimum  and the maximum  of nullcline 1, which corresponds to the positivity of the element  in the Jacobian matrix .

In two-component ODE systems, a single negative feedback loop is not sufficient for sustained oscillations. Additionally, one of the two components must activate itself autocatalytically ([50] and [6, Chapter 9]). This results in two different kinds of two-dimensional oscillators, the *activator-inhibitor oscillator* (AIO) and the *substrate-depletion oscillator* (SDO). Both are characterized by the course of their nullclines and, related to that, the signed Jacobian matrix  at the unstable fixed point  in the interior of the limit cycle, which is (6)

for the AIO and the SDO, respectively. Examples for these two oscillator models can be found in [6, 26, 27]. A typical course of the nullclines for an AIO is shown in Figure 1. Note that both the AIO and the SDO do not strictly fulfill the monotonicity condition. Due to the nonlinear autocatalytic activation, the sign of the corresponding diagonal element in  depends on the state , as can be seen at nullcline 1 in the figure. This is, however, not a problem here, since relevant statements still hold if the state space is partitioned into regions in which the signs of the Jacobian matrix are constant [2]. Moreover, in many biological networks, a self-regulation is often not a direct interaction, but comprises intermediate components. An example is a protein that promotes expression of its own gene as a transcription factor. Here, strict monotonicity can again be reconstructed by introducing two separate variables for mRNA and protein concentration, respectively.

A stable limit cycle surrounds an unstable fixed point that is given as the intersection of the two nullclines. A fixed point  can only be unstable if it is located between the minimum  and the maximum  of nullcline 1. This corresponds to a positive element  at the fixed point . It has been shown that this condition becomes sufficient for sufficiently large time-scale differences [26, 27]. Furthermore, it is not required any more when including time delays, which was exemplarily shown for a specific AIO model in [27].

Here, we show that a stable fixed point  with signed Jacobian matrix of the forms  or (6) can indeed *always* be destabilized through a time delay . This result is an extension of the previous results described in [27], where we proved that a bifurcation via an increase in the time delay always destabilizes a fixed point. Furthermore, this destabilization is caused by a Hopf bifurcation and therefore creates a stable limit cycle. Thus,  becomes as well a sufficient condition for the existence of a stable limit cycle in AIO models, provided that the time delay is sufficiently large. This is stated in the following Theorem 1. The proof is given in Appendix A. We remark here that this theorem can analogously be proven for SDO models.

Theorem 1 (instability of  through a time delay). 

Assume system (2) to have a stable fixed point  for  and signed Jacobian matrix  as given in (6). An increase in the time delay  eventually destabilizes .

Theorem 1 implies that there exists a threshold time delay  such that  is unstable if . 

Corollary 1. 

If system (2) has a stable fixed point  for  and , there exists a threshold time delay  such that  is unstable for . The proof is given in Appendix B

According to Theorem 1 and Corollary 1, if an AIO model has a single fixed point that lies between the minimum  and the maximum  of the first nullcline (Figure 1), this fixed point is unstable for a sufficiently large time delay .

**Figure 2:**
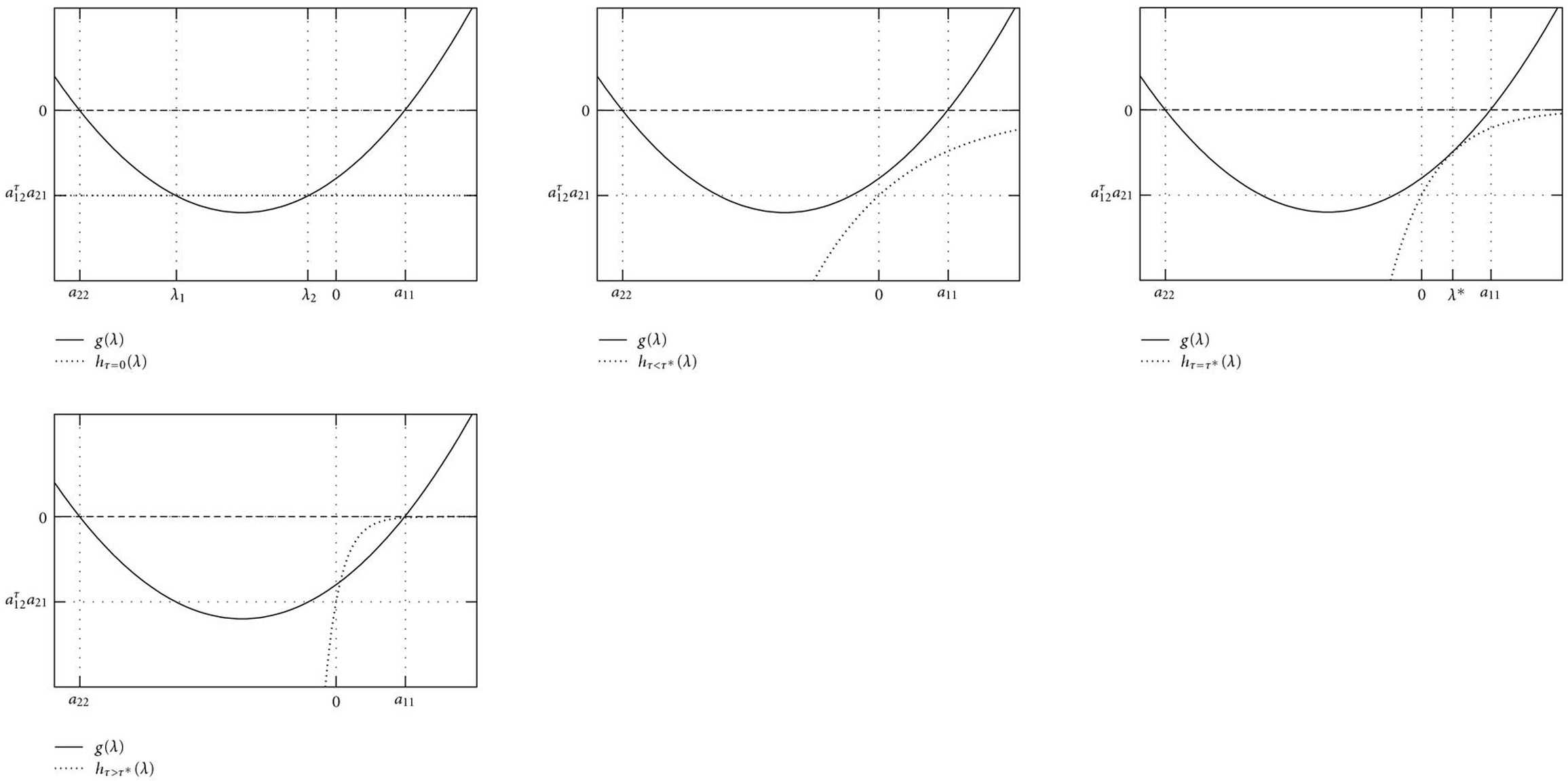
**Eigenvalues  of the Jacobian matrix  correspond to intersections of the two function  and .** This Figure illustrates how a stable fixed point is destabilized by a time delay . (a) For ,  is a constant function, and the Jacobian matrix  has two negative real eigenvalues  and . (b) For , the function  is a strictly increasing function that approaches 0 exponentially. Thus, increasing , the two real eigenvalues coalesce to a pair of complex conjugate eigenvalues, whose real parts eventually become positive. (c) A further increase in  leads to a new osculation point  of  and  at a value . This  is positive and real, and hence corresponds to an unstable fixed point. (d) For , the two eigenvalues approach the values  and .

**Figure 3:**
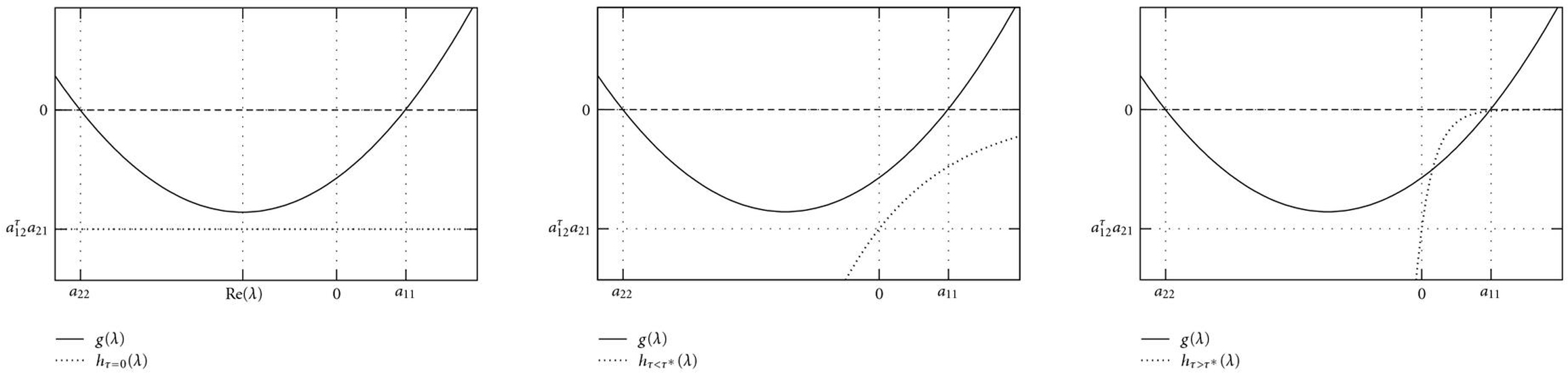
(a)  has a pair of complex conjugate eigenvalues with negative real parts. (b) The real part of this pair increases with increasing time delay . (c) A further increase in  eventually leads to positive real eigenvalues, as already demonstrated in Figure 2.

**Figure 4:**
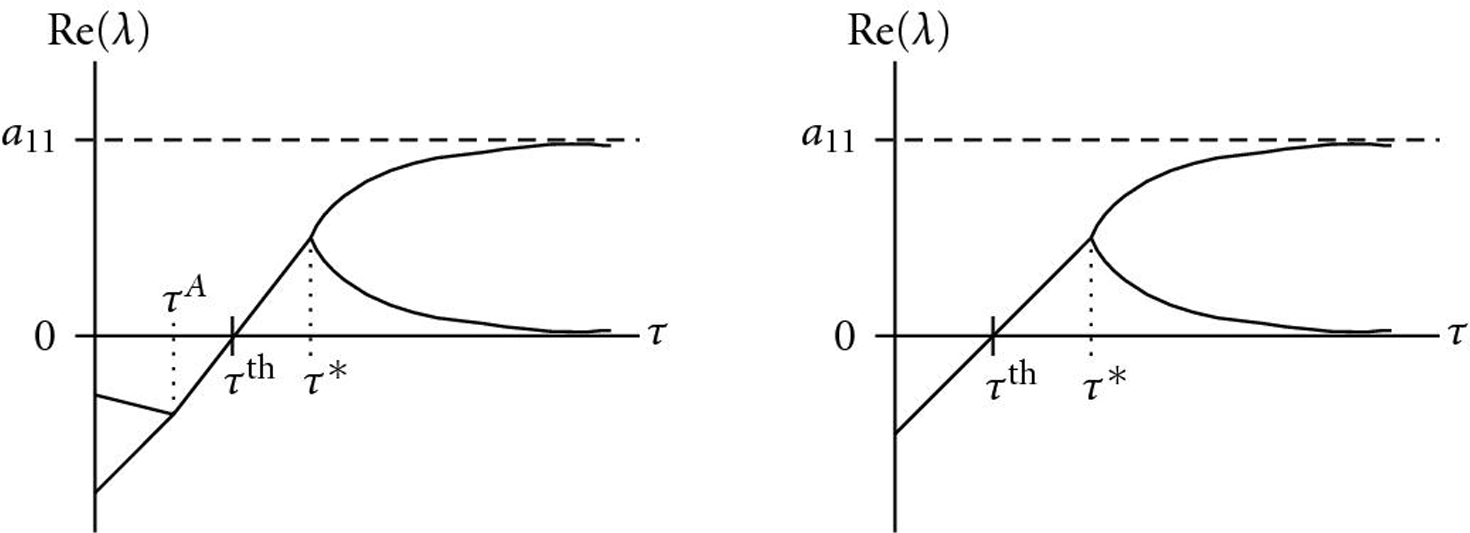
Schematic change of the real parts of the two eigenvalues of an originally stable fixed point . (a)  has two negative real eigenvalues (compare also Figure 2). At , they coalesce to a pair of complex conjugate eigenvalues whose real part increases with . The system undergoes a Hopf bifurcation and the fixed point  becomes unstable when this real part crosses the -axis at . For large time delays, the two eigenvalues are real from  and approach the values 0 and . (b) Similar course, , has a pair of complex conjugate eigenvalues.

Figures 2, 3, and 4 illustrate how a stable fixed point  of an AIO is destabilized by a time delay . According to (5), eigenvalues  can graphically be interpreted as (probably complex) â€œintersectionsâ€� of the two functions  and  (Figures 2 and 3). A sketch of the real parts of the two eigenvalues  as a function of  is shown in Figure 4. For Figure 2, we have used the functions  and , that is, parameter values ,  and  in (5). With these parameters, the Jacobian matrix  has two negative real eigenvalues  and . Increasing , these eigenvalues eventually coalesce at a value  (Figure 4(a)) and become a pair of complex conjugates, whose real parts increase with . The fixed point  becomes unstable through a Hopf bifurcation when the real part crosses the -axis at . Further increasing , the imaginary parts vanish again at , and both real eigenvalues eventually approach  and , respectively. In Figure 3,  was changed to â€“350, and  has a pair of complex conjugate eigenvalues  with negative real parts. The behavior of their real parts is shown in Figure 4(b) and similar to the course in Figure 4(a).

**Figure 5:**
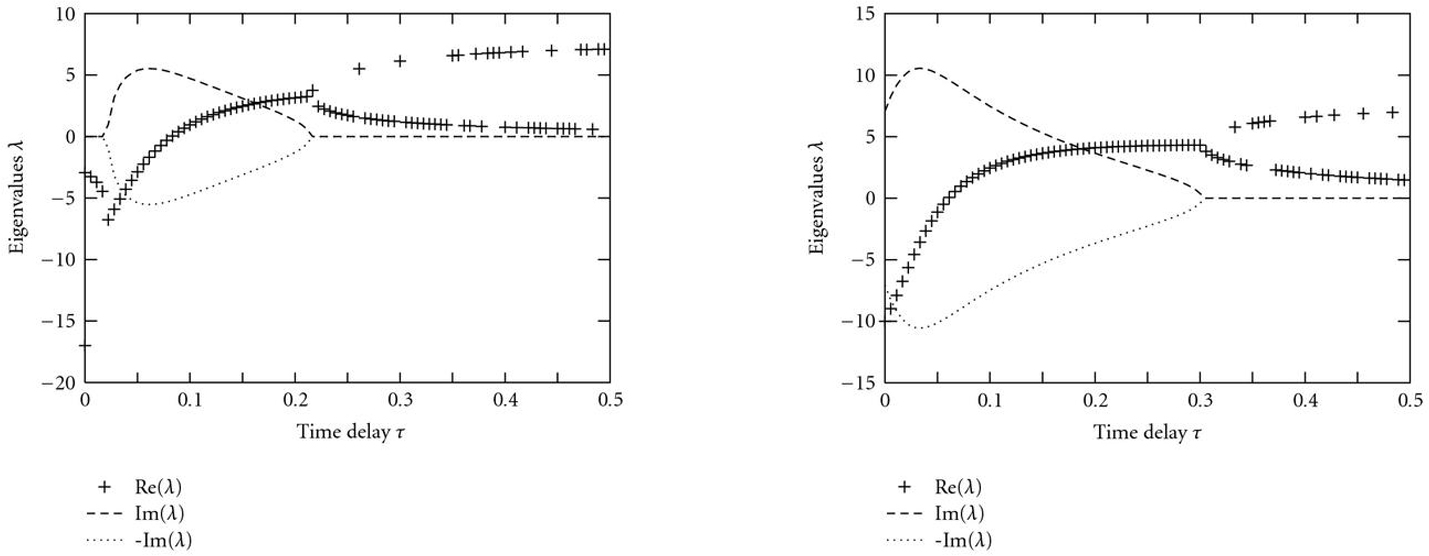
**Solutions  of (5) as a function of the time delay  for parameter values , , and  (a) and  (b).** The equation was solved with the Newton method and random initial starting points.

We checked this result also numerically by separating real and imaginary parts of (5) and solving for  and . This was done using the Newton method iteratively for a delay interval . Resulting real and imaginary parts are shown in Figure 5. The course is in agreement with that in Figure 4. The two eigenvalues for  are . Initial guesses  were set using a Monte Carlo approach. Both eigenvalues present and built a pair of complex conjugates with increasing real parts. Finally, the imaginary part vanishes again and  and  approach  and , as described before. Simulation studies (not shown here) indicate that the two eigenvalues  investigated here are the rightmost ones of the spectrum, since  is exactly destabilized when they cross the imaginary axis.

The inclusion of a sufficiently large time delay  like in (2) destabilizes  via a Hopf bifurcation. This guarantees the existence of a stable limit cycle at least around the bifurcation value . Moreover, in case that  is the only fixed point of the system, destabilizing  implies global convergence to a stable limit cycle from arbitrary initial conditions. This follows from the PoincarÃ©-Bendixson theorem (PBT), which states that the -limit set of a bounded forward trajectory of a two-dimensional system is either a steady-state or a limit cycle [45]. In other words, a bounded solution of such a system either converges to a fixed point or to a limit cycle.

### 2.3. Generalizations for Higher Dimensions

**Figure 6:**
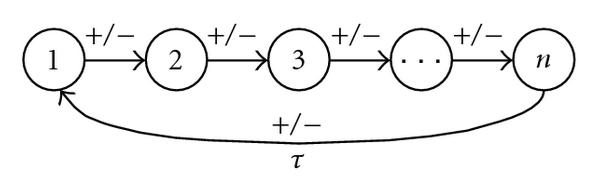
**Single negative feedback loop with a time delay  for the regulation of  onto .** Signs of edges are arbitrary except that their product has to be negative.

The analysis of systems with more than two variables can be more complex, since concepts of the phase plane analysis and related theorems can not always directly be transferred to the , . An example is the PBT, which is often used to show the existence of a stable limit cycle in two-dimensional systems. There is no general analogous theorem for higher dimensions. At least, the Hopf bifurcation theorem [44] claims the existence of such a limit cycle locally about the bifurcation parameter. However, some extensions of the PBT to differential equation systems whose flow is on a two-dimensional manifold [45] and regulatory systems of more than two components and special graph structures have been considered [51, 52]. Among these systems is the single negative loop structure, which I consider in the following. It consists of a single negative feedback loop (Figure 6). The product of edge signs has to be negative. We include a time delay in the regulation of variable  to variable . The corresponding system of differential equations is given by(7)

with monotonicity and boundedness constraints as specified in Section 2. GouzÃ© [2] has shown that vector fields of regulatory systems with interaction graphs lacking positive circuits are injective, which implies that they have at most one fixed point. The existence of such a fixed point for the single negative loop system is shown in [53]. All together, this system has a unique fixed point, and according to [51], a stable limit cycle exists in case that this fixed point is unstable. This allows once again for a reduction of the analysis of the whole system onto the stability of its fixed point via a linearization about  and the spectrum of .

The following theorem generalizes results in [27], where the same statement was proven for two-dimensional systems. 

Theorem 2. 

A Hopf bifurcation caused by an increase of the time delay  in system (7) always destabilizes a stable fixed point. The pair of complex conjugate eigenvalues crosses the imaginary axis from left to right, (8)

The proof can be found in Appendix C.

Theorem 2 shows that whenever an increase in the time delay  causes a Hopf bifurcation in the single loop system, this bifurcation destabilizes a stable fixed point and creates a limit cycle.

However, sufficient conditions for the occurrence of such a bifurcation in negative loop systems similar to that in two-dimensional systems remain to be investigated in this context. Also for the single loop system, the introduction of a positive autoregulation of one of the components seems to be sufficient for the existence of such a threshold value, with the same convergence argument as for two-dimensional systems. Unlike in two-dimensional systems, however, it is not clear for arbitrary network structures whether the destabilization of a fixed point implies the existence of a stable limit cycle not only locally in the neighborhood of a bifurcation.

## 3. On the Impact of Bifurcations for the Inverse Problem

In this section, the effect of bifurcations on the inverse problem to estimate parameters from time series data is investigated. We consider the following inverse problem. Given a differential equation model  and time series data  the task is to estimate values for the parameter vector  by minimizing an objective function : (9)

Optimization problems of this kind are important for all fields in which differential equations are used to describe dynamic behaviors, and model parameters are to be adapted to experimental data. For nonlinear systems, these problems are known to be difficult to solve, since the surface of the objective function  has some undesirable properties. Efficient optimization algorithms are required in order to obtain reliable estimates within an acceptable time. Several approaches have been proposed (see, e.g., [39] and the subsequent discussions for an overview, or [37, 38] for a method called â€œmultiple shootingâ€� and applications to biological systems). Generally, these optimization problems will become even more important in systemsâ€™ biology in the future.

Here we show the impact of bifurcations on the objective function  with a special focus on bifurcations relevant for periodic behavior in regulatory systems. Results are illustrated using the AIO model described in [27]: (10)(11)

with parameter vector . The true vector is given by . The system has a globally stable limit cycle for these values.

We investigate two objective functions , first, the sum of squared errors between measurements  and model predictions : (12)

and second, the sum of differences between  and , weighted by the time difference ,(13)

In the limit , (13) corresponds to the area between the two â€œcurvesâ€�  and . Values for  are numerically calculated by a simple Euler discretization, and initial values are set to . By the way, numerical integration has to be performed in each step of a gradient-based optimization approach and is usually the limiting factor concerning computing time.

Of course, even without noise, (12) and (13) depend on the dataset , in particular, on the initial vector  and the sampling time points. Here, we show exemplary examples for fixed initial conditions  and simulations over the transient and two oscillation periods. Further, for simplicity reasons, we vary only one single parameter  at a time, the *control parameter*, while the rest is fixed to the true values . Thus, the measurements  are obtained via simulations using , and  corresponds to simulations using  and a different value for the control parameter . In order to overcome the dependence of the error functions (12) and (13) on the sequence of sampling time points, we use a very small time step , which corresponds to 10.000 Euler steps for numerical integration in the simulated range.

### 3.1. Nonsmoothness

**Figure 7:**
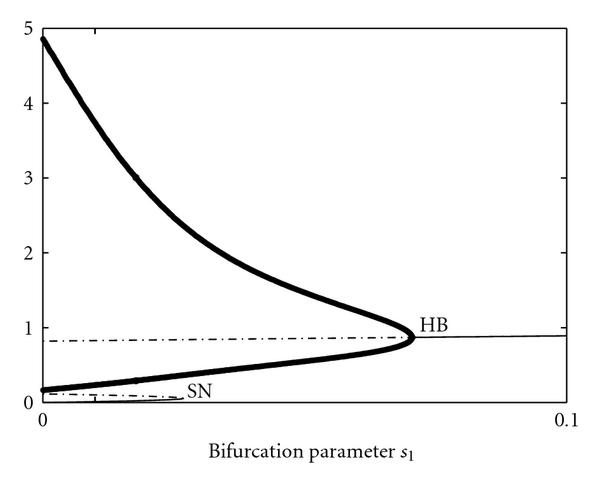
Bifurcation diagram of system (11) and bifurcation or control parameter .

**Figure 8:**
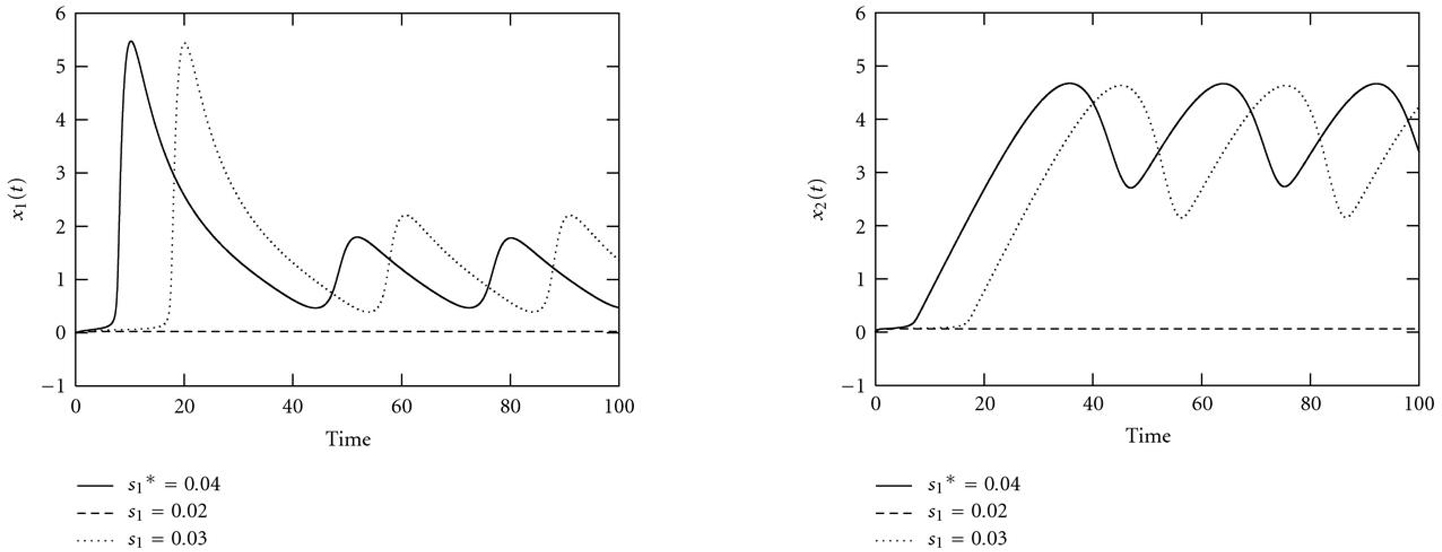
Courses of (a)  and (b)  for different control parameter values  along with  and initial values . For ,  is in the basin of attraction of the lower stable fixed point in Figure 7. If  exceeds the saddle node bifurcation, this leads to a change of the qualitative dynamic behavior of , which approaches the globally stable limit cycle for  SN (here illustrated by ).

Figure 7 shows the bifurcation diagram of system (11) with control parameter . All bifurcation plots were created with the program xppaut [54]. For , the system has two separated -limit sets, a stable limit cycle and a stable fixed point. Increasing , the stable fixed point coalesces with an unstable one in a saddle-node (SN) bifurcation, and the limit cycle becomes globally attracting. Finally, the system undergoes a supercritical Hopf bifurcation (HB), in which the second unstable fixed point becomes globally stable and the limit cycle vanishes. For a value  SN and initial conditions , the system converges to the lower stable fixed point. The difference between the two curves  and  is large, as shown in Figure 8 and control value . Increasing , both objective functions  and  remain almost constant. Near the bifurcation value SN, a slight increase of  causes an abrupt change in the qualitative dynamic behavior from convergence to oscillations (). This goes along with a jump in the objective function at the saddle-node bifurcation, which reaches zero at the true value  and increases smoothly thereafter.

Such jumps in the error function are generally related to bifurcations at which stable -limit sets disappear, typically saddle-node or subcritical Hopf bifurcations in our context. As a consequence, gradient-based methods might be much more efficient when the step size is adapted during the gradient descent to optimize .

### 3.2. Local Suboptimal Minima

Figure 10 shows the bifurcation diagram with control parameter . For low values of , the system has a stable limit cycle around an unstable fixed point. This limit cycle vanishes at a saddle-node bifurcation (SN), where an unstable and a globally stable fixed point emerges. While the dependence of the -coordinate of this fixed point is only marginal (Figure 11(a)), the coordinates of  increase with increasing  (Figure 11(b)), which leads to a local suboptimal minimum in the error functions, here at a value  (Figure 12). Moreover, it can be seen that the true value  has a relatively small basin of attraction, which is bounded by the saddle-node bifurcation (SN). On the contrary, the basin of attraction for the local minimum at , which corresponds to the converging time series, is much larger.

Hence, starting a local search method with an arbitrary initial parameter vector leads in most cases to suboptimal minima which correspond to systems that converge to a stable fixed point. These local minima render global search methods such as simulated annealing or genetic algorithms necessary, which usually require long running times [40]. Thus, efficient algorithms are needed in this context.

### 3.3. Ruggedness Near the True Parameter Value

Figure 13 shows the bifurcation diagram with control parameter . The system has a stable limit cycle bounded by two supercritical Hopf bifurcations (HBs). Within this oscillating region, period and amplitude vary considerably with the control parameter, as indicated in the simulations in Figure 14. This dependency causes multiple local minima in the error functions (Figure 15). Thus, even if the optimization process is already started within the oscillating region in the parameter space, a simple gradient search might fail to find the true parameter value but get stuck in one of the local minima.

This emphasizes again the necessity of efficient optimization algorithms for parameter estimation of differential equation models in general.

## 4. Conclusions

This paper investigated the robustness of sustained oscillations in regulatory systems with respect to varying model parameters. Differential equations based on chemical reaction kinetics, which are often used for this purpose, are not always robust, and oscillations only occur in a small region of the parameter space bounded by bifurcation manifolds.

In the first part of the paper, we focused on the inclusion of time delays into the differential equations. Time delays take some time between the cause and the effect of a regulation into account, and they are known to stabilize oscillations by enlarging the region in the parameter space which correspond to periodic solutions. Since the typical behavior of the class of systems considered here is convergence to a fixed point, oscillations are usually induced via destabilizing a fixed point through a Hopf bifurcation. We investigated the stability of a fixed point in dependence of the time delay. We provided sufficient conditions for a time delay to induce oscillations in two-dimensional systems, in particular, activator-inhibitor oscillator and substrate-depletion oscillator models, which are the typical oscillator models in two dimensions. These conditions are graphically defined in terms of the qualitative course of the nullclines, which are usually easily accessible. Specifically, if the system has a single fixed point located between the minimum and the maximum of one of the nullclines, it can always be destabilized by a sufficiently large time delay, which implies sustained oscillations. Results are based on rather general assumptions about the underlying differential equation system, which hold for many related oscillator models.

Moreover, for single-loop systems with an arbitrary number of components we showed that a Hopf bifurcation that is caused by increasing the time delay always destabilizes a stable fixed point. The real parts of the eigenvalues of the Jacobian matrix at the fixed point change signs from negative to positive. Thus, a stable fixed point can loose stability by increasing the time delay, which leads to the existence of a stable limit cycle, but an unstable fixed point cannot become stable.

Here, the analysis of the system was done by linearizing the system about a fixed point and investigating the stability of this fixed point via the spectrum of eigenvalues. This facilitates the analysis of the long-term behavior considerably. We referred to the conditions necessary for such a destabilization of a fixed point to imply sustained oscillations. The PoincarÃ©-Bendixson theorem is extremely useful in this context for two-dimensional systems. Similar theorems exist for higher-dimensional systems with special interaction graphs. The single-loop system considered here belongs to these systems. However, for a further generalization of these results to higher dimensional systems, the following questions remain to be investigated in the future. First, can the class of systems that have a unique fixed point be further characterized? It is already known that networks lacking a positive feedback loop have at most one fixed point. Second, how can this be further generalized to networks that also contain positive feedback circuits? Networks with only positive loops cannot have stable limit cycles. Consequently, oscillations can only occur in networks that have at least one negative loop. Contrary to negative feedback control, positive loops can lead to multiple fixed points. Hence for such â€œmixed-circuit networks,â€� it is not sufficient any more to show the existence of a fixed point. It also has to be investigated whether it is unique. However, necessary conditions for multiple fixed points in positive loop systems are also known to be very restrictive, and many of these models seem to have a unique fixed point, too. Third, in which cases does a destabilization of a fixed point lead to oscillating behavior? And forth, what are sufficient conditions for the existence of a threshold time delay ?

The second part of this work investigated the influence of bifurcations on the inverse problem to estimate model parameters from time series data. Such bifurcations are generally related to nonsmoothness and multiple local minima of the objective function to be optimized in this setting. Although these phenomena are generally not new, the focus was on the special properties occurring in the class of oscillating models considered here. Global search methods are required to find the real optimum. Together with the numerical integration, these methods are usually extremely time-consuming, even for small systems with only a few parameters.

In a realistic setting, the problem is even worse. First, the optimization problem is of course multidimensional. All values of model parameters have in principle to be found at a time, which renders a comprehensive search and an investigation of the whole objective function difficult. Second, the data is usually noisy and sparse, leading to ill-posed optimization problems. Noisy datasets also mean that the measured initial condition  might not always be the best choice for . Generally,  should be included into the objective function as an additional variable that has to be optimized as well, which increases the dimension of the inverse problem even further. Moreover, the dataset might also contain missing values or unobserved variables. This raises additional problems, and estimating parameters by minimizing the residual error might fail in this context anyway. In this setting, stochastic approaches might be more convenient, since they take the noise in the dataset into account. Bayesian learning approaches, for example, allow for an appropriate regularization via prior distributions over model parameters. Concluding, the development of efficient approaches for parameter estimation in differential equation models remains a challenging research field in the future.

## Appendices

### A. Proof of Theorem 1

We prove this statement by showing that one of the eigenvalues of the Jacobian matrix  approaches the positive element  in the limit , that is,  or, equivalently, . The function  in 5 is a parabola with two zeros  and , . Furthermore,(A1)

This implies  and hence , which implies further that  is an eigenvalue of  in the limit . Consequently,  cannot be stable.

### B. Proof of Corollary 1

**Figure 9:**
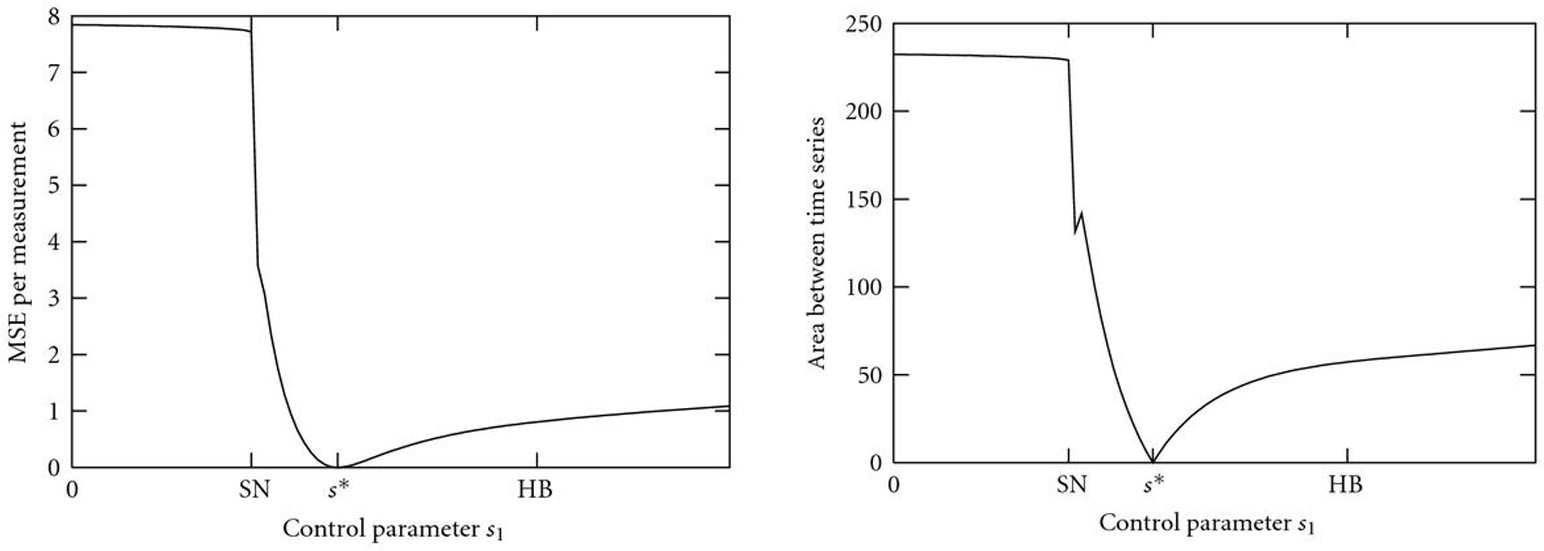
** (a)  and (b)  versus control parameter .** Below the saddle-node bifurcation (SN), both values are almost constant. The SN causes a jump in both error functions. Since the Hopf bifurcation (HB) is supercritical, the error changes only smoothly around this value.

We use the following two statements for AIOs, which are derived in [27].

(1)The real part of an eigenvalue  of the Jacobian matrix  is a continuous function of , .

(2)Moreover, a sign change of the real part of an eigenvalue  caused by an increase of the time delay  is always a change from positive to negative, and includes a pair of complex conjugate eigenvalues  with nonzero imaginary parts , that is, . Increasing , both objective functions  and , which are shown in Figure 9, remain almost constant.

**Figure 10:**
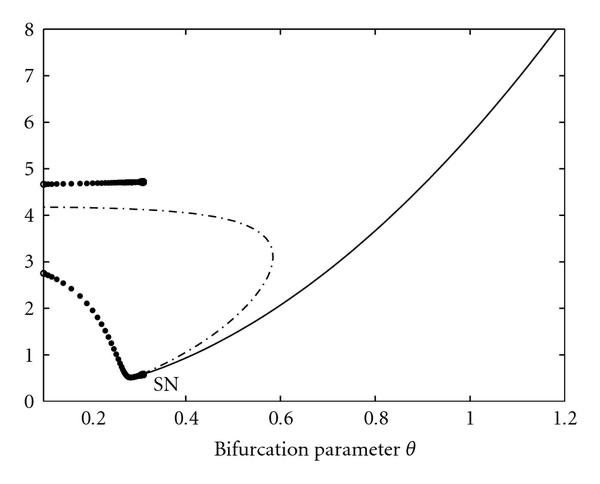
Bifurcation diagram of system (11) with bifurcation parameter .

**Figure 11:**
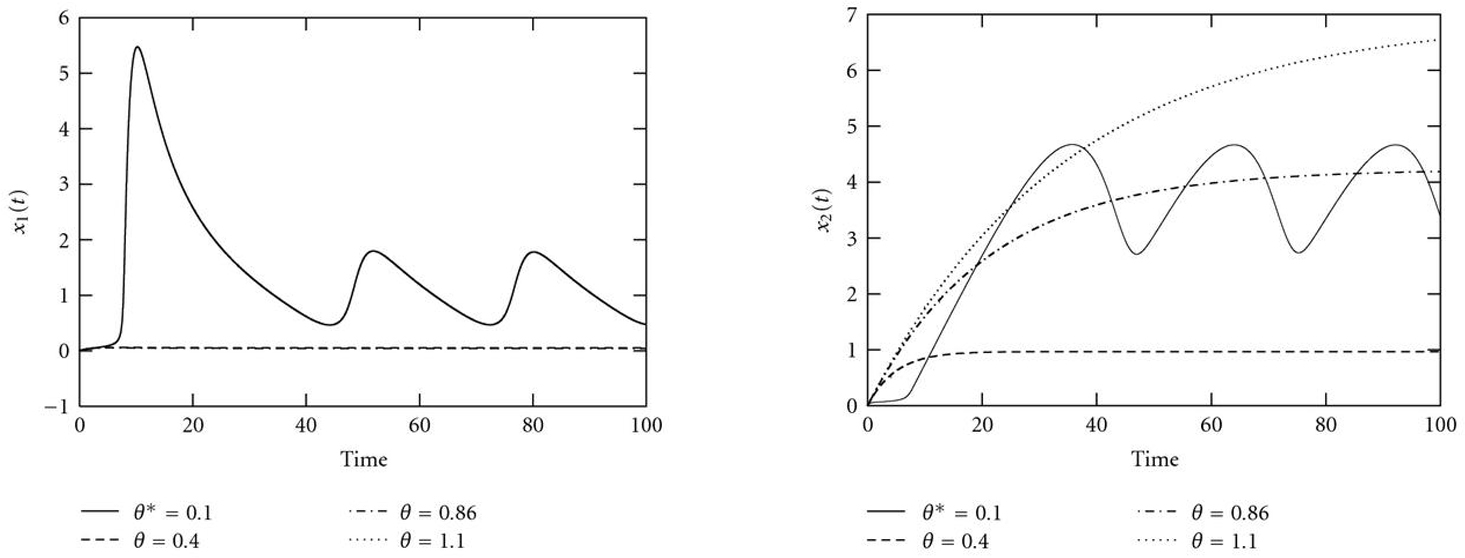
Courses of (a)  and (b)  for varying control parameters . The system shows periodic behavior for the real value  and converges to a globally stable fixed point for all other values shown. The coordinates of this stable fixed point remain low for  (the courses cannot be distinguished in this plot), and increase with increasing  for . This causes a smooth local minimum at  in the error function (Figure 12).

**Figure 12:**
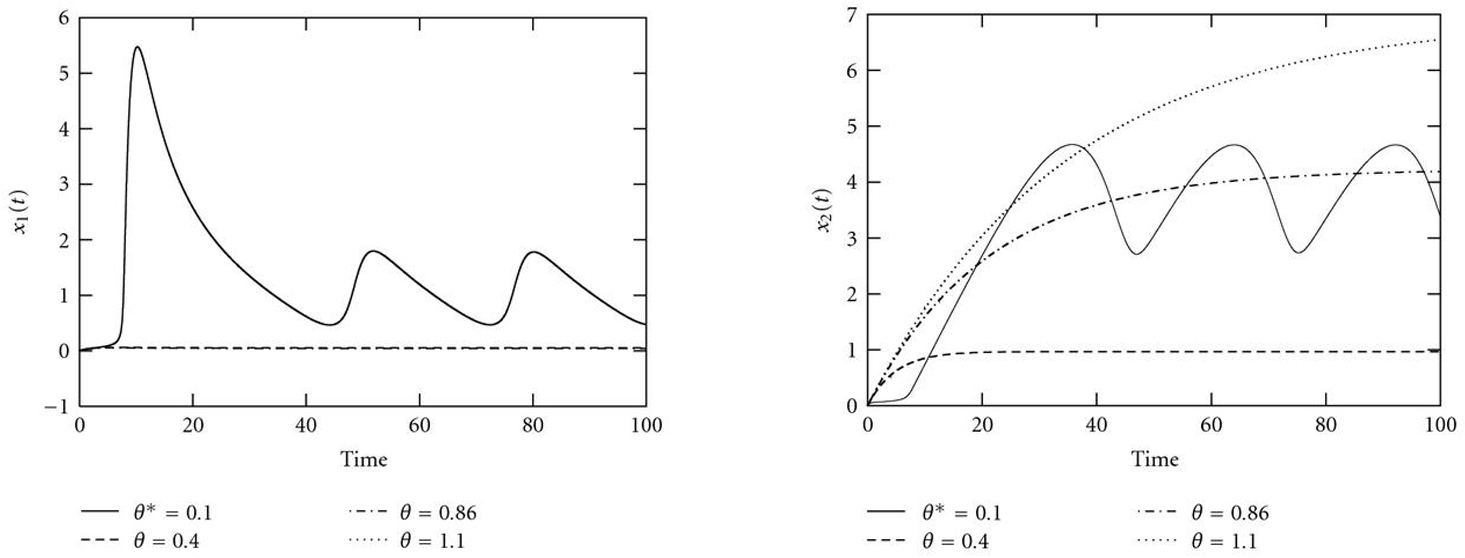
** (a)  and (b)  versus control parameter .** The true value  has a relatively small basin of attraction, which is bounded by a saddle-node bifurcation (SN). The basin of attraction for the local minimum at , which corresponds to a system with globally stable fixed point, is much larger.

**Figure 13:**
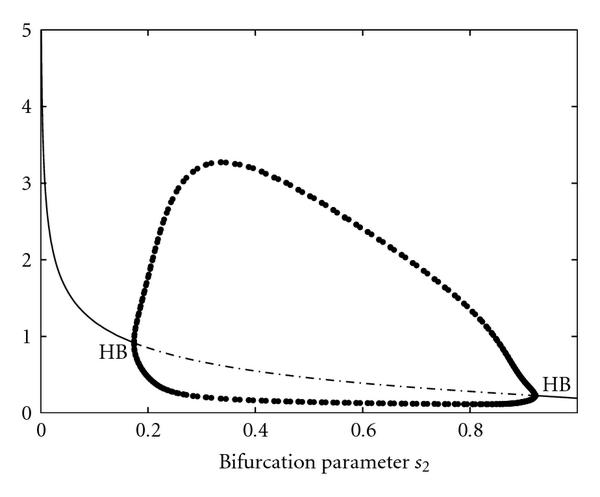
Bifurcation diagram for system (11) with bifurcation parameter .

**Figure 14:**
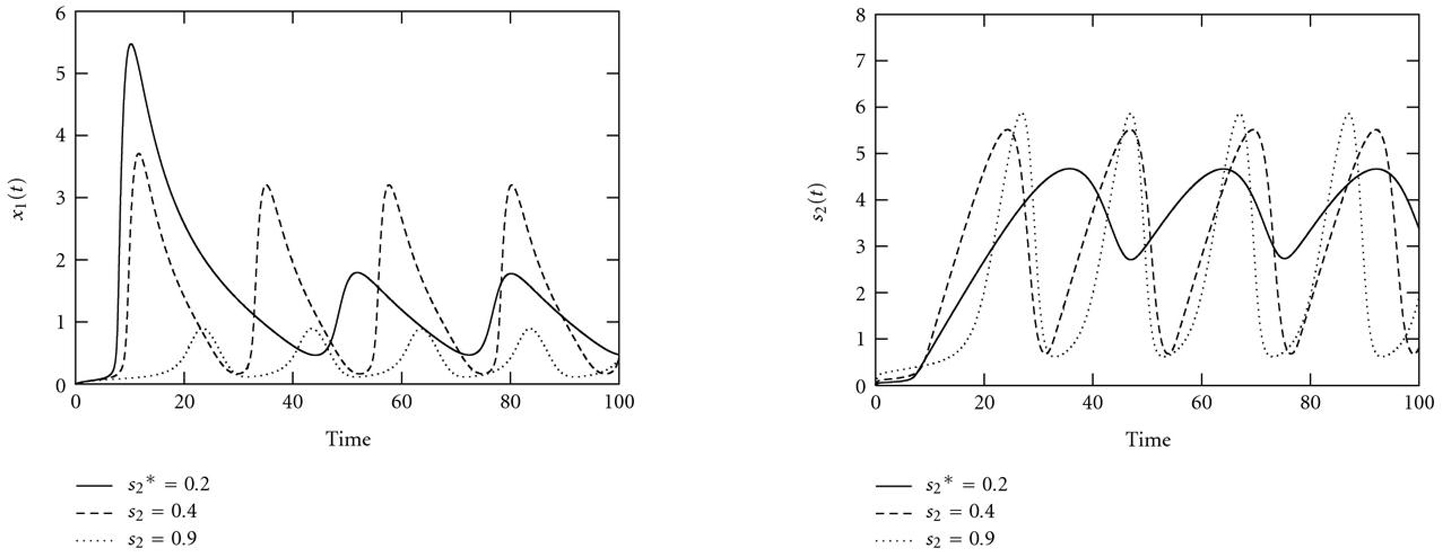
Courses of (a)  and (b)  for different values of the control parameter , which have been chosen between the two Hopf bifurcations in Figure 13. Both amplitude and period of the oscillations vary with varying control parameter value, which causes several local minima in the error function (Figure 15).

**Figure 15:**
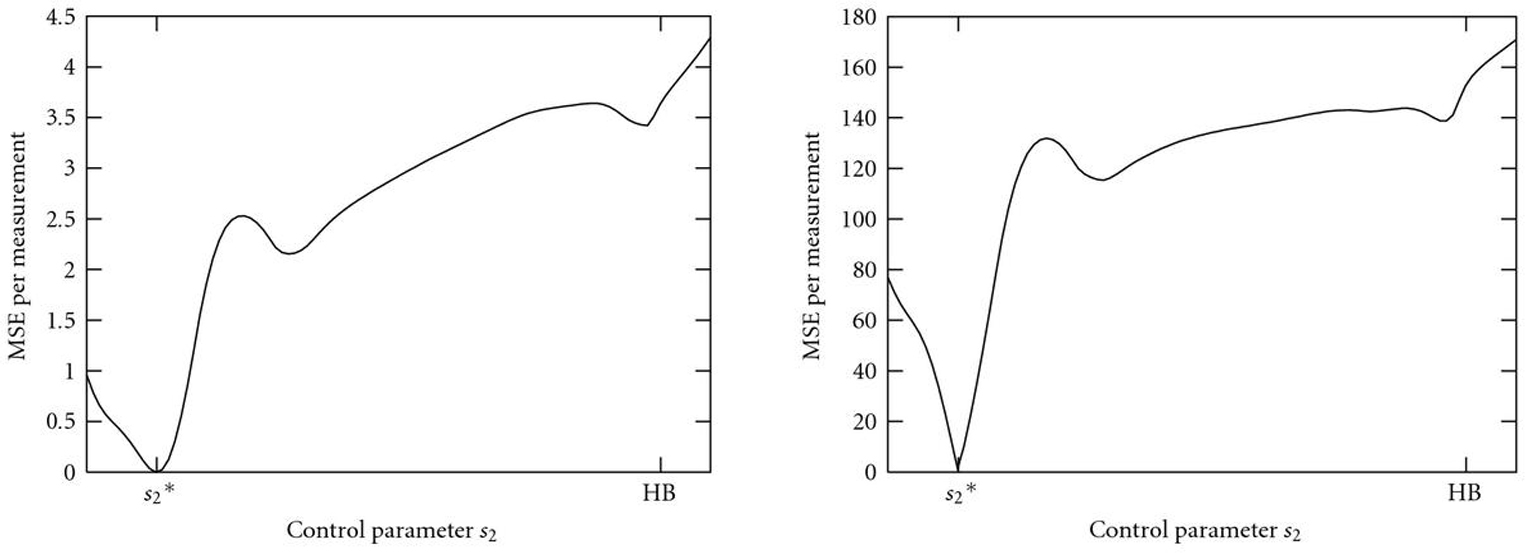
**(a)  and (b)  versus control parameter .** The dependence of the amplitude and especially the period of the oscillations on the control parameters cause several local minima in both error functions.

Statement (1) allows to apply the intermediate value theorem (IVT). Starting with a stable fixed point  for  whose real parts of the eigenvalues of  are negative,  has at least one zero according to the IVT and Theorem 1. Uniqueness of this zero follows from statement 2, since a second zero would be a transition from positive to negative, that is, , a contradiction.

### C. Proof of Theorem 2

Linearizing system 7 about , the characteristic equation is(C1)

The equation  has to be solved. For this, it is convenient to take the logarithm of both sides, which gives the condition(C2)

Calculating the derivatives of both sides with respect to  leads to(C3)

Resolving for  yields(C4)

Here it is worth noting that although  is a real number, the derivative  is complex. It can be understood as the inverse of the derivative , which is defined by the condition(C5)

We consider the real parts of both sides in C.4: (C6)

with (C7)

Inserting  and resolving for  show that this derivative is positive for purely imaginary eigenvalues :(C8)
